# Multilevel analysis of covariation in socioeconomic predictors of physical functioning and psychological well-being among older people in rural Vietnam

**DOI:** 10.1186/1471-2318-10-7

**Published:** 2010-02-11

**Authors:** Hoang Van Minh, Dao Lan Huong, Stig Wall, Nguyen Thi Kim Chuc, Peter Byass

**Affiliations:** 1 Faculty of Public Health, Hanoi Medical University Vietnam, Hanoi, Vietnam; 2 Health Strategy and Policy Institute, Hanoi, Vietnam; 3 Umeå International School of Public Health, Umeå, Sweden

## Abstract

**Background:**

There remains a lack of research on co-variation of multiple health outcomes and their socio-economic co-patterning, especially among the elderly. This papers aims to 1) examine the effects of different socio-economic factors on physical functioning and psychological well-being among older adults in a rural community in northern Vietnam; and 2) investigate the extent to which the two outcomes variables co-vary within individuals.

**Methods:**

We analyzed the data from the WHO/INDEPTH study on global ageing and adult health conducted on 8535 people aged 50 years old and over in Bavi district of Vietnam in 2006. A multivariate response model was constructed to answer our research questions. The model treats the individual as a level two unit and the multiple measurements observed within an individual as a level one unit.

**Results:**

Lower physical functioning and psychological well-being were found in 1) women; 2) older people; 3) people with lower education level; 4) people who were currently single; 5) respondents from poorer household; and 6) mountainous dwellers compared to that in those of other category(ies) of the same variable. Socioeconomic factors accounted for about 24% and 7% of variation in physical functioning and psychological well-being scores, respectively. The adjusted correlation coefficient (0.35) indicates that physical functioning and psychological well-being did not strongly co-vary.

**Conclusions:**

The present study shows that there exist problems of inequality in health among older adults in the study setting. This finding highlights the importance of analyzing multiple dimensions of health status simultaneously in inequality investigations.

## Background

The past century has witnessed a demographic transition characterized by a rapid ageing process. Population projections for the coming years show a considerable increase in the proportion of older people worldwide [1-3]. To effectively and efficiently respond to the growing health needs of elderly populations, it is critical to have in-depth understandings about their health conditions and related socioeconomic factors.

Literature from developed countries has consistently shown that socioeconomic disparities in health existed in general populations as well as among the elderly. Individuals with lower socioeconomic positions are more likely to suffer from both morbidity and mortality [4,5]. Similarly, elders who had higher education and socioeconomic status experienced less depression [6]. However, studies from developing countries have shown inconsistent findings on the effects of socioeconomic status on elderly health. Some studies show that older adults in lower socio-economic positions generally experience worse health than those in better-off groups [7-11]. Some other studies showed that the strength of associations between socioeconomic status and elderly health were less clear or non-existent [12,13].

Although many studies have been conducted to examine inequalities in health status across socioeconomic groups, almost all of them have investigated different aspects of health status separately [14]. There remains lack of research on co-variation of multiple health outcomes and their socio-economic co-patterning, especially among the elderly. As "health" is defined by WHO as "a state of complete physical, mental and social well-being and not merely the absence of disease or infirmity" [15], it is important to explore co-morbidity of physical functioning and psychological well-being among the elderly.

Vietnam, a developing country in South-East Asia, has experienced the population aging phenomenon. The proportion of people aged 50 years and over rose from 12.6% in 2000 to 14.1% in 2005 and will account for 18.9% of total population in 2015 [3]. Similar to other low-and middle-income countries, little research has been conducted in Vietnam on the issues of elderly health, particularly in regard to the co-variation of different health outcomes. In response to the necessity and urgency of having scientific evidence on the issues of socioeconomic determinants of elderly health, this paper, using a multivariate multilevel modelling approach, aims to 1) examine the effects of different socio-economic factors on physical functioning and psychological well-being among older adults in a rural community in northern Vietnam; and 2) investigate the extent to which the two outcome variables co-vary within individuals.

## Methods

### Data source

We used data from the WHO/INDEPTH study on global ageing and adult health. The overall study, conducted during 2006-2007, gathered information on health state and quality of life among people aged 50 years old and over in nine demographic surveillance sites from the International Network for the continuous Demographic Evaluation of Populations and Their Health in developing countries (INDEPTH) http://www.indepth-network.net: South Africa (Agincourt and Hlabisa), Viet Nam (Filabavi), Tanzania (Ifakara), Bangladesh (Matlab), Kenya (Nairobi), Ghana (Navrongo), Indonesia (Purworejo), and India (Vadu). The total sample size was over 46,000 respondents [16].

In Vietnam, this study was conducted in 2006 in a rural district in Northern Vietnam, Bavi district, within the framework of a Demographic Surveillance System called FilaBavi (Epidemiological Field Laboratory of Bavi). Face-to-face household interviews were conducted with all the people aged 50 years old and over who lived in FilaBavi areas. The interviews were done by trained surveyors of the FilaBavi using standard WHO/INDEPTH questionnaire- summary version. More detailed descriptions of the Bavi district and FilaBavi can be found elsewhere [17].

## Measurements

### Outcome variables

In this study, both physical functioning and psychological well-being were analysed as outcome variables. Physical functioning was measured by asking respondents about their functional difficulties in the last 30 days, including the level of difficulty 1) in standing for long periods; 2) in taking care of your household responsibilities; 3) in joining in community activities [for example, festivities, religious or other activities] in the same way as anyone else can; 4) in concentrating on doing something for 10 minutes; 5) in walking a long distance such as a kilometre; 6) in washing (bathing) the whole body; 7) in getting dressed; and 8) in day to day work. For psychological well-being, the study subjects were asked: 1) "Overall in the last 30 days, how much of a problem did you have with feeling sad, low or depressed?" and 2) "Overall in the last 30 days, how much of a problem did you have with worry or anxiety?"

The response set for each question was a five-point scale where 1 = None, 2 = Mild, 3 = Moderate, 4 = Severe, 5 = Extreme/cannot do. The scale reliability coefficient (Cronbach's alpha) for physical functioning and psychological well-being questions was 0.89 and 0.86, respectively. Total score for each dimension (i.e. physical functioning and psychological well-being) was the sum of all the relevant question scores. Higher scores indicated a person with poorer status of physical functioning or psychological well-being.

### Independent variables

We included a wide range of socioeconomic information as independent variables such as sex, age, educational level, marital status of the individual, household size, place of residence and economic status of the household. Educational level was categorized into three groups: I: No schooling; II: Less than six year of education; III: Graduated from primary school and higher. Marital status was categorized as: I: Currently in marital partnership (living with spouse or partner); II: Currently single (never married, divorced or widowed). Place of residence was defined I: Riverside/island; II: Highland; and III: Mountainous area. Economic status of the respondent's household was measured by asset-based wealth quintiles. The wealth quintiles were constructed using a principal component analysis technique [18].

### Statistical analysis

**Figure 1:**
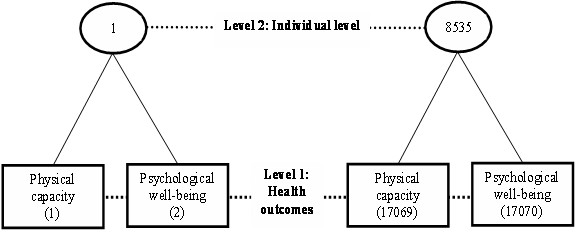
**Multilevel structure**.

A multivariate response model was constructed to answer our research questions. The model treats the individual as a level two unit and the multiple measurements observed within an individual as a level one unit. We developed a 2-level model of 17070 (two outcomes for each individual) at level 1 nested within 8535 individuals at level 2 (Figure 1). By treating multiple outcomes within the multivariate response model, we were able to estimate the covariance between two outcomes nested within individuals, as well as the variance for each outcome in a simultaneous manner.

The two-level model can be written as follows:

Where:

Z_2ij _= 1 - Z _lij_,

X_j _= independent variables (gender, age, marital status, education, wealth status, place of residence, household size),

var(U_1j_) = σ^2^_u1 _= variance in physical functioning,

var(U_2j_) = σ^2^_u2 _= variance in psychological well-being,

cov(U_1j _U_2j_) = σ_u12 _= covariance of physical functioning and psychological well-being

The modeling was done in two steps. In Model 1 (Empty model), no explanatory variable was included. Model 2 (Full model) had all independent variables. The results were presented as coefficient and standard errors (SE). A significance level of p < 0.05 was used. MLwiN software, version 2.02 http://www.cmm.bristol.ac.uk/MLwiN, was used for the analyses.

### Ethical considerations

The protocol of this study was approved by the Scientific Board of the FilaBavi. All human subjects in the study were asked for their written informed consent before collecting data, and all had complete right to withdraw from the study at any time without having any threat or disadvantage.

## Results

### Characteristics of the study populations

Of the total of 8,874 people aged 50 years and over who lived in the study setting at the time of the survey, there were 8,535 who participated in the study (96%). 4% did not respond to the survey because they were away (2.3%) or were not healthy enough to take part in the survey (1.7%). There were no significant differences in socioeconomic characteristics between the respondents and the non-respondents. Of the final sample, 37.7% were aged 50-59 years, 36.5% 60-69 years, 24.4% 70-79 years and 11.4% 80 years and over. The characteristics of the final sample are described in Table 1.

**Table 1:** Characteristics of the study participants, FilaBavi, Vietnam 2006

Characteristics	50-59 n [%]	60-69 n [%]	70-79 n [%]	80+ n [%]	All ages n [%]
**Gender**					
- Male	1436 [44.6]	1023 [45.3]	757 [36.3]	253 [26.1]	3469 [40.6]
- Female	1785 [55.4]	1235 [54.7]	1329 [63.7]	717 [73.9]	5066 [59.4]
**Education**					
- No schooling	56 [1.7]	110 [4.9]	298 [14.3]	414 [42.7]	878 [10.3]
- Less than six year of education	940 [29.2]	1245 [55.1]	1493 [71.6]	512 [52.8]	4190 [49.1]
- Graduated from primary school and higher	2225 [69.1]	903 [40.0]	295 [14.1]	44 [4.5]	3467 [40.6]
**Marital status**					
- Currently in marital partnership	2772 [86.1]	1714 [75.9]	1152 [55.2]	257 [26.5]	5895 [69.1]
- Currently single	449 [13.9]	544 [24.1]	934 [44.8]	713 [73.5]	2640 [30.9]
**Socio-economic**					
- 1st quintile (poorest)	277 [8.6]	285 [12.7]	467 [22.4]	180 [18.6]	1209 [14.2]
- 2nd quintile	523 [16.3]	436 [19.4]	432 [20.8]	157 [16.2]	1548 [18.2]
- 3rd quintile	694 [21.6]	510 [22.6]	405 [19.5]	178 [18.4]	1787 [21.0]
- 4th quintile	853 [26.6]	545 [24.2]	375 [18.0]	223 [23.0]	1996 [23.4]
- 5th quintile (richest)	866 [27.0]	477 [21.2]	403 [19.4]	230 [23.8]	1976 [23.2]
**Place of residence**					
- Riverside/island	1113 [34.6]	753 [33.4]	702 [33.7]	355 [36.6]	2923 [34.3]
- Highland	1600 [49.7]	1165 [51.6]	991 [47.5]	443 [45.7]	4199 [49.2]
- Mountainous area	508 [15.8]	340 [15.1]	393 [18.8]	172 [17.7]	1413 [16.6]

**Total**	**3221 [100]**	**2258 [100]**	**2086 [100]**	**970 [100]**	**8535 [100]**

### Levels of physical functioning and psychological well-being

The scores of physical functioning ranged from 5 to 40. The psychological well-being levels were between 2 to 10. The means (and the corresponding standard deviations) of physical functioning and psychological well-being levels reported by the study respondents are presented in Table 2.

**Table 2:** Levels of physical functioning and psychological well-being among older adults, FilaBavi, Vietnam 2006.

Socioeconomic variables	Physical functioning mean [sd]	Psychological well-being mean [sd]
**Gender:**		
- Male	13.7 [6.7]	3.4 [1.9]
- Female	15.5 [7.0]	3.8 [2.2]
**Age:**		
50-59	11.9 [4.9]	3.5 [2.0]
60-69	13.7 [5.9]	3.7 [2.1]
70-79	16.9 [7.2]	3.7 [2.2]
80+	22.1 [7.9]	3.8 [2.2]
**Education:**		
- No schooling	20.6 [8.1]	4.2 [2.4]
- Less than six year of education	15.5 [6.9]	3.7 [2.1]
- Graduated from primary school and higher	12.4 [5.5]	3.4 [1.9]
**Marital status:**		
- Currently in marital partnership	13.6 [6.2]	3.5 [2.0]
- Currently single	17.5 [7.7]	4.0 [2.3]
**Socio-economic:**		
- 1st quintile [poorest]	16.3 [7.3]	4.2 [2.4]
- 2nd quintile	15.1 [7.0]	3.8 [2.1]
- 3rd quintile	14.7 [6.8]	3.6 [2.0]
- 4th quintile	14.4 [7.0]	3.5 [2.0]
- 5th quintile [richest]	14.1 [6.7]	3.3 [1.9]
**Place of residence:**		
- Riverside/island	15.6 [7.2]	3.8 [2.1]
- Highland	15.2 [6.8]	3.3 [1.8]
- Mountainous area	14.1 [6.8]	3.6 [2.1]

The variations in physical functioning and psychological well-being levels by socio-demographic variables were similar. Lower physical functioning and psychological well-being were found in 1) women; 2) older people; 3) people with lower education level; 4) people who were currently single; 5) respondents from poorer household; and 6) mountainous dwellers compared to those in other category(ies) of the same variable.

### Multilevel modelling results

**Figure 2:**
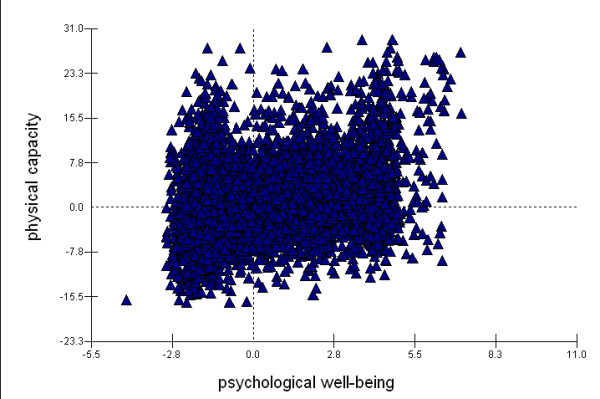
**Correlation between physical functioning and psychological well-being**. Correlation coefficient = 0.35, after adjusting for socio-economic variables.

Table 3 shows the random part of the multivariate response model. The empty model indicates that there was significant variation in each outcome of interest. The variance in both physical functioning and psychological well-being became smaller after the socio-economic variables were included (Full model). The socioeconomic factors accounted for about 24% and 7% of variation in physical functioning and psychological well-being scores, respectively. There was a significant correlation between these two independent variables. About 14% of the covariance between these two outcomes of interest was attributable to the socioeconomic factors. However, the adjusted correlation coefficient of 0.35 in the full model indicates that physical functioning and psychological well-being did not strongly co-vary (i.e. an individual with poorer physical functioning did not necessarily have a lower level of psychological well-being and vice versa) (Figure 2).

**Table 3:** Random part of the multivariate response model, FilaBavi, Vietnam 2006

	Empty model		Full model		Change in estimate
	Estimate	SE	Estimate	SE	
Variance in Physical capacity	4.4	0.06	4.1	0.06	7%
Variance in Psychological well-being	48.4	0.74	36.6	0.56	24%
Covariance	5.0	0.17	4.3	0.14	14%

Table 4 presents the fixed part findings of the full model. There were some similarities as well as differences in socioeconomic patterning of physical functioning and psychological well-being. Women were more likely to have poor physical functioning and low psychological well-being levels than men. While age was shown to be a negative predictor of physical functioning, it had no significant effect on psychological well-being. People with higher educational levels reported being better in both physical functioning and psychological well-being. Those who were currently in marital partnerships had better status of physical functioning and psychological well-being. Significant economic differentials were found for psychological well-being (i.e. the better-off had less psychological problems) but not for physical functioning. Mountainous dwellers had significantly lower levels of both physical functioning and psychological well-being. Household size had no important effect on the two outcomes variables.

**Table 4:** Fixed part of the multivariate response model, FilaBavi, Vietnam 2006

Socioeconomic variables	Physical capacity Coefficient [SE]	Psychological well-being Coefficient [SE]
**Gender:**		
- Male	Reference	Reference
- Female	0.34 [0.16]*	0.2 [0.05]*
**Age:**		
50-59	Reference	Reference
60-69	1.48 [0.17]*	0.08 [0.06]
70-79	4.05 [0.20]*	-0.02 [0.07]
80+	8.40 [0.27]*	-0.16 [0.09]
**Education:**		
- No schooling	Reference	Reference
- Less than six year of education	-2.20 [0.24]*	-0.35 [0.08]*
- Graduated from primary school and higher	-2.88 [0.29]*	-0.46 [0.10]*
**Marital status:**		
- Currently in marital partnership	Reference	Reference
- Currently single	0.58 [0.17]*	0.26 [0.06]*
**Socio-economic:**		
- 1st quintile [poorest]	Reference	Reference
- 2nd quintile	0.02 [0.24]	-0.34 [0.08]*
- 3rd quintile	-0.08 [0.24]	-0.48 [0.08]*
- 4th quintile	-0.16 [0.24]	-0.52 [0.08]*
- 5th quintile [richest]	-0.42 [0.25]	-0.72 [0.08]*
**Family size:**	0.02 [0.04]	-0.02 [0.01]
**Place of residence:**		
- Riverside/island	-0.61 [0.20]*	-0.58 [0.07]*
- Highland	-1.42 [0.15]*	-0.24 [0.05]*
- Mountainous area	Reference	Reference
		
**Intercept:**	15.15 [0.38]*	4.54 [0.13]*

* denotes a significant result (p < 0.05)

## Discussion

In this study, adopting the WHO's definition of health, we considered both physical functioning and psychological well-being as fundamental end points of elderly health. Taking advantage of the multilevel modelling approach, we were able to investigate the co-variation in socioeconomic correlates of physical functioning and psychological well-being within individuals.

We found some common significant socioeconomic predictors of both physical functioning and psychological well-being among the elderly in the study setting such as gender, education and place of residence. Independently of other factors, women were shown to suffer more from both physical and psychological problems. This is consistent with findings from previous studies in Asia which showed that women were more likely to reported poor health than men [19-21]. A strong positive effect of education on both physical functioning and psychological well-being was also pronounced. Studies from Japan [7], Taiwan [8] and China [9] also reported that higher educational attainment resulted in a decreased incidence of functional limitations. We found that mountainous dwellers had lower levels of physical functioning and psychological well-being compared to people living in other areas. Negative effects of disadvantage residence on elderly health were also revealed in a Chinese study [22], and a Korean-Japanese study [23].

We also observed that while physical functioning declined with advancing age, psychological well-being did not vary significantly by age. Similar observation was documented in study in the US [14] and in China [24].

In the present study, economic differentials were found for psychological well-being but not for physical functioning. The overwhelming age effect possibly diluted the influence of economic status on physical functioning. Studies from Thailand [12] and Taiwan [25] showed that income had significant independent influences on functional disorders. A study from US found strong income associations for both health and happiness [14].

It is worth noting from the multivariate response model that the socioeconomic variables of interest had stronger associations with physical functioning (accounting for 24% of variation) than psychological well-being (accounting for 7% of total variation). As a result, the two health outcomes did not co-vary to a strong degree (adjusted correlation coefficient 0.35). This finding highlights the importance of analyzing multiple dimensions of health status simultaneously in inequality investigations. Examining a single health outcome may misclassify a person as being in "good health" while in fact he/she has another bad outcome. The single health outcome approach may also lead to underestimation of inequality problems in a population.

There are several limitations we need to note from this study. Firstly, accuracy and validity of self-reported information in older people could be questionable. Low educational level and the presence of cognitive retardation in older people might have reduced the accuracy and validity of the findings. Secondly, because of the cross-sectional nature of the data, our study could not provide any interpretation of causal relations between socioeconomic status and physical functioning and psychological well-being among the elderly in the study setting. Thirdly, some possible joint effects of socio-economic factors on the outcome variables have not been investigated.

## Conclusions

The present study provides initial insight into the extent to which socioeconomic indicators are related to multiple health outcomes among older adults in a developing country setting. It shows that there exist problems of inequality in health among older adults in the study setting. The evidence should be useful for health authorities in responding to the growing health-related needs of elderly populations with limited economic resources. Investigation of health issues among older people is not simple task. This multilevel modelling approach, which offers several technical advantages, should be further utilized. More sophisticated research, such as using longitudinal study designs, is needed to examine the causal relationship between multiple health outcomes and socioeconomic conditions.

## Competing interests

The authors declare that they have no competing interests.

## Authors' contributions

HVM, DLH, PB, SW made substantial contributions to conception and design, or acquisition of data, or analysis and interpretation of data. HVM, DLH, SW, NTKC and PB have been involved in drafting the manuscript or revising it critically for important intellectual content. All authors read and approved the final manuscript.

## Pre-publication history

The pre-publication history for this paper can be accessed here:

http://www.biomedcentral.com/1471-2318/10/7/prepub

## References

[B1] Omran AR The epidemiological transition theory revisited thirty year latter World health statistic quart 1998 51 99 119

[B2] World Health Organization Active aging: A policy framework 2002 Geneva World Health Organization

[B3] United Nations World Population Prospects: The 2006 Revision and World Urbanization Prospects 2006 http://esa.un.org/unpp

[B4] Mackenbach JP Kunst AE Cavelaars AEJM Groenhof F Geurts JJM Socioeconomic inequalities in morbidity and mortality inwestern Europe The Lancet 1997 349 9066 1655 1659 10.1016/S0140-6736(96)07226-1 9186383

[B5] Knesebeck Ovd Lüschen G Cockerham WC Siegrist J Socioeconomic status and health among the aged in the United States and Germany: A comparative cross-sectional study Social Science & Medicine 2003 57 9 1643 1652 10.1016/S0277-9536(03)00020-0 12948573

[B6] Fiske A Wetherell JL Gatz M "Depression in older adults" Annu Rev Clin Psychol 2009 5 363-89 10.1146/annurev.clinpsy.032408.153621 PMC2852580 19327033

[B7] Liu X Liang J Muramatsu N Sugisaea H Transitions in functional status and active life expectancy among older people in Japan Journal of Gerontology 1995 50 S383 394 10.1093/geronb/50b.6.s383 7583817

[B8] Zimmer Z Liu X Hermalin A Chuang YL Educational attainment and transitions in functional status among older Taiwanese Demography 1998 35 3 361 363 10.2307/3004043 9749327

[B9] Liang J Liu X Gu S Transitions in functional status among older people in Wuhan, China: Socioeconomic differentials Journal of Clinical Epidemiology 2001 54 11 1126 1138 10.1016/S0895-4356(01)00390-0 11675164

[B10] Berkman CS Gurland BJ The relationship among income, other socioeconomic indicators, and functional level in older persons J Aging Health 1998 10 1 81 98 10.1177/089826439801000105 10182419

[B11] Zimmer Z Kwong J Socioeconomic Status and Health among Older Adults in Rural and Urban China J Aging Health 2004 16 1 44 70 10.1177/0898264303260440 14979310

[B12] Zimmer Z Amornsirisomboon P Socioeconomic status and health among older adults in Thailand: an examination using multiple indicators Social Science & Medicine 2001 52 8 1297 1311 10.1016/S0277-9536(00)00232-X 11281411

[B13] Zimmer Z Chayovan N Lin H-S Natividad J How Indicators of Socioeconomic Status Relate to Physical Functioning of Older Adults in Three Asian Societies Research on Aging 2004 26 2 224 258 10.1177/0164027503260624

[B14] Subramanian SV Kim D Kawachi I Covariation in the socioeconomic determinants of self rated health and happiness: a multivariate multilevel analysis of individuals and communities in the USA J Epidemiol Community Health 2005 59 8 664 669 10.1136/jech.2004.025742 16020643 PMC1733107

[B15] World Health Organization Preamble to the Constitution of the World Health Organization International Health Conference. NewYork 1946

[B16] World Health Organization, INDEPTH network Study on Global Ageing and Adult Health (SAGE) 2006 http://www.who.int/healthinfo/systems/sage/en/index2.html

[B17] Chuc NTK Diwan VK FilaBavi, a demographic surveillance site, an epidemiological field laboratory in Vietnam Scand J Public Health 2003 31 Suppl 62 3 7 10.1080/14034950310015031 14578073

[B18] Vyas S Kumaranayake L Constructing socio-economic status indices: how to use principal components analysis 2006 21 459 468 17030551 10.1093/heapol/czl029

[B19] Ahmad K Jafar T Chaturvedi N Self-rated health in Pakistan: results of a national health survey BMC Public Health 2005 5 1 51 10.1186/1471-2458-5-51 15943882 PMC1164420

[B20] Rahman MO Barsky AJ Self-Reported Health Among Older Bangladeshis: How Good a Health Indicator Is It? Gerontologist 2003 43 6 856 863 14704385 10.1093/geront/43.6.856

[B21] Lim W-Y Ma S Heng D Bhalla V Chew S Gender, ethnicity, health behaviour & self-rated health in Singapore BMC Public Health 2007 7 1 184 10.1186/1471-2458-7-184 17655774 PMC1976324

[B22] Liu G Zhang Z Sociodemographic Differentials of the Self-rated Health of the Oldest-old Chinese Population Research and Policy Review 2004 23 2 117 133 10.1023/B:POPU.0000019921.20777.1b

[B23] Lee Y Shinkai S A comparison of correlates of self-rated health and functional disability of older persons in the Far East: Japan andKorea Archives of Gerontology and Geriatrics 2003 37 1 63 76 10.1016/S0167-4943(03)00021-9 12849074

[B24] Qin H Adding life to years: predicting subjective quality of life among Chinese oldest-old Master thesi 2007 College of Arts and Sciences, Georgia State University

[B25] Chiu HC Hsieh YH Mau LW Lee ML Associations between socio-economic status measures and functional change among older people in Taiwan Ageing & Society 2005 25 377 395 10.1017/S0144686X05003478

